# Prognostic value of pulmonary diffusing capacity for carbon monoxide and ventilation–perfusion SPECT findings in pulmonary arterial hypertension

**DOI:** 10.1113/EP091688

**Published:** 2024-05-09

**Authors:** Milan Mohammad, Jacob P. Hartmann, Jørn Carlsen, Anders M. Greve, Ronan M. G. Berg, Jann Mortensen

**Affiliations:** ^1^ Centre for Physical Activity Research Copenhagen University Hospital – Rigshospitalet Copenhagen Denmark; ^2^ Department of Biomedical Sciences, Faculty of Health and Medical Sciences University of Copenhagen Copenhagen Denmark; ^3^ Department of Clinical Physiology and Nuclear Medicine Copenhagen University Hospital – Rigshospitalet Copenhagen Denmark; ^4^ Department of Cardiology Copenhagen University Hospital Rigshospitalet Copenhagen Denmark; ^5^ Department of Clinical Medicine, Faculty of Health and Medical Sciences University of Copenhagen Copenhagen Denmark; ^6^ Department of Clinical Biochemistry Copenhagen University Hospital Rigshospitalet Copenhagen Denmark; ^7^ Neurovascular Research Laboratory, Faculty of Life Sciences and Education University of South Wales Pontypridd UK; ^8^ Department of Medicine The National Hospital Torshavn Faroe Islands

**Keywords:** haemoglobin, lung diffusing capacity, lung function, mortality, prognosis, pulmonary vascular disease

## Abstract

Reduced pulmonary diffusing capacity for carbon monoxide (D_LCO_) can be observed in pulmonary arterial hypertension (PAH) and associates with increased mortality. However, the prognostic value of D_LCO_ when corrected for haemoglobin (D_LCOc_), an independent modifier of D_LCO_, remains understudied. Additionally, the prognostic role of ventilation (V)–perfusion (Q) emission computed tomography (V/Q SPECT) findings in patients with PAH, which may concurrently be performed to rule out chronic thromboembolic pulmonary hypertension, is uncertain. A retrospective cohort study was conducted on 152 patients with PAH referred to a tertiary hospital for evaluation from January 2011 to January 2020. Lung function tests, clinical data and V/Q SPECT were ascertained. Cox regression analysis was performed to evaluate the association between D_LCOc_, D_LCO_ and V/Q SPECT defects at referral with all‐cause mortality. In equally adjusted Cox regression analysis, each percentage increase in D_LCOc_ % predicted (%pred) (hazard ratio (HR) 0.97; 95% CI: 0.94–0.99) and D_LCO_%pred (HR 0.97; 95% CI: 0.94–0.99) was similarly associated with all‐cause mortality. There was no detectable difference in area under the curve for prediction of all‐cause mortality by D_LCOc_%pred and D_LCO_%pred (C‐index 0.71 and 0.72, respectively, *P* = 0.85 for difference). None of the defects noted on V/Q SPECT were significantly associated with mortality, but mismatched defects were associated with lower values of D_LCOc_%pred and D_LCO_%pred. D_LCOc_%pred and D_LCO_%pred perform equally as prognostic markers in PAH, supporting the use of either metric when available for prognostic stratification.

## Abstract (highlights)

- **What is the central question of this study?**
  Does haemoglobin correction of the pulmonary diffusing capacity for carbon monoxide improve prognostic stratification in patients with pulmonary arterial hypertension (PAH)?
- **What is the main finding and its importance?**
  In PAH, the pulmonary diffusing capacity for carbon monoxide predicts all‐cause mortality equally well regardless of whether it is haemoglobin‐corrected or not. Either metric can therefore be used for the diagnostic workup of these patients. Ancillary findings on ventilation–perfusion emission computed tomography concurrently performed to rule out chronic thromboembolic pulmonary hypertension did not inform prognosis further.

## 1 INTRODUCTION

Pulmonary hypertension (PH) is a progressive disorder of the pulmonary circulation with a persistent >20 mmHg increase in mean pulmonary arterial pressure at rest (Galiè et al., 2019; Humbert et al., 2022). It is a heterogenous disease, which according to the European Society of Cardiology (ESC) and the European Respiratory Society (ERS) guidelines is classified in five diagnostic groups of which Group 1 encompasses pulmonary arterial hypertension (PAH), a pathological condition that can manifest because of diverse aetiological factors. In terms of pathophysiology, PAH is distinguished by conspicuous histological modifications as evidenced by vascular remodelling occurring within the pulmonary vascular bed and a notable augmentation in pulmonary vascular resistance (Humbert et al., 2022; Tuder, Stacher et al., 2013). This encompasses augmented contractility of pulmonary arterioles, endothelial dysfunction, disrupted signalling pathways and changes in endothelial and smooth muscle cells (Tuder, Archer et al., 2013).

Patients with PAH are haemodynamically characterised by pre‐capillary PH in the absence of other causes of pre‐capillary PH, such as chronic thromboembolic pulmonary hypertension (CTEPH) or lung disease. Therefore, for appropriate classification and prognostic stratification of PH, patients undergo extensive diagnostic workup to investigate the presumed predominant cause of increase in pulmonary pressure. This includes the measurement of pulmonary diffusing capacity for carbon monoxide (D_LCO_) and ventilation–perfusion single‐photon emission computed tomography (V/Q SPECT) to rule out signs of CTEPH.

D_LCO_ relies on the inhalation and transfer of carbon monoxide to the haemoglobin (Hb) within the pulmonary capillaries. Therefore, it may be diminished in PAH due to changes in the pulmonary vasculature (Sun et al., 2003). A low baseline D_LCO_ has previously been reported to be independently associated with adverse outcomes and increased mortality over a 1, 3 and 5‐year period (Benza et al., 2010; Chandra et al., 2010; Diamanti et al., 2022; Hamada et al., 2007; Lefèvre et al., 2013; Stadler et al., 2019), and a D_LCO_ of less than 45% of predicted is now used to define a specific PAH subphenotype (Hoeper et al., 2022). However, since D_LCO_ is markedly affected by blood haemoglobin (MacIntyre et al., 2005; Tsai et al., 2013) and CO–Hb binding is pivotal in CO transfer, these findings may be confounded by the presence of anaemia, polycythaemia and erythrocytosis (Dumitrescu et al., 2017), of which anaemia is prevalent among most PH patients and is associated with poor clinical outcomes, increased disease severity, morbidity and mortality (Krasuski et al., 2011; Sonnweber et al., 2020). Still, the decrease in D_LCO_ primarily suggests involvement of the alveolar–capillary membrane rather than anaemia per se. At present, it remains to be investigated whether correcting D_LCO_ for haemoglobin in accordance with current clinical guidelines leads to better prognostication.

Whilst computed tomography (CT) pulmonary angiography is the method of choice to diagnose CTEPH (PH Group 4) in patients with PH, this may be preceded by V/Q SPECT, which can effectively rule out the presence of pulmonary thromboembolic disease, that is, by the absence of characteristic segmental or subsegmental perfusion defects (Bajc et al., 2019). V/Q SPECT patterns can be classified as matched defects (both perfusion and ventilation defects at the same anatomical site), inversely mismatched ventilation defects (only a defect in ventilation) and mismatched perfusion defects (only a defect in perfusion). However, a separate identifiable pattern with global patchy changes and heterogeneous perfusion defects on the V/Q SPECT images is often observed in PAH (Chan et al., 2018; Wang et al., 2019). This pattern is associated with reduced D_LCO_ (Mohammad et al., 2021) as perfusion defects affect diffusing capacity negatively, and has been suggested to be indicative of a poor prognosis (Auger et al., 2012; Chan et al., 2018; Lang et al., 2010). Nonetheless, the precise significance of these varying defects on V/Q SPECT in the prognostic assessment of patients with PAH, who had no detectable thromboembolism observed during corroborating investigations, remains indeterminate.

In this retrospective cohort study, we tested the hypotheses (1) that haemoglobin‐correction of D_LCO_ (D_LCOc_) leads to better prediction of all‐cause mortality than D_LCO_; and (2) that findings from V/Q SPECT scans provide supplementary prognostic information during the diagnostic workup of PH.

## 2 METHODS

### 2.1 Ethical approval

The study conformed to the standards set by the *Declaration of Helsinki*, except for registration in a database. The study was approved by the Danish Patient Safety Authority and the Danish Data Protection Agency (file no. 31‐1521‐236). In accordance with Danish legislation, additional approval was not required from the Regional Scientific Ethical Committee for this retrospective study, nor was additional informed consent from patients required, apart from that already provided for having V/Q SPECT and lung function tests performed as part of the clinical workup of PH.

### 2.2 Study design and setting

This single‐centre retrospective cohort study is reported according to the Strengthening the Reporting of Observational Studies in Epidemiology (STROBE) Statement (von Elm et al., 2007). We evaluated medical records from all patients with PH verified by right heart catheterisation in the Department of Cardiology and subsequently referred to the Department of Clinical Physiology and Nuclear Medicine at Rigshospitalet in Copenhagen between January 2011 and February 2020.

### 2.3 Participants

All available patients referred for PH‐related lung function testing as a part of the diagnostic workup between 1 January 2011 and 28 February 2020 were identified in our local lung function database at the Department of Clinical Physiology and Nuclear Medicine at Rigshospitalet in Copenhagen, Denmark. The final PH diagnosis was assessed through the PH database (DAN‐PH) at the Department of Cardiology, Rigshospitalet in Copenhagen. Only patients with an available lung function and/or V/Q SPECT from PH Group 1 (PAH) were included with no conflicting co‐morbidities that caused the increased pulmonary pressure. Patients belonging to other aetiological PH groups (Groups 2–5) were not included, including those with mismatched perfusion defects on V/Q SPECT in whom thromboembolic disease was subsequently diagnosed on CT pulmonary angiography.

### 2.4 Variables

#### 2.4.1 Lung function testing

All lung function tests were performed at the Department of Clinical Physiology and Nuclear Medicine, Rigshospitalet by trained personnel in a standardised manner in accordance with international guidelines (MacIntyre et al., 2005; Miller et al., 2005; Wanger et al., 2005) as a part of the initial assessment. This was done with Jaeger MasterScreen PFT pro system (CareFusion, Höchberg, Germany), and included dynamic spirometry, whole‐body plethysmography and single‐breath uptake of CO. The following data were obtained: forced expiratory volume in 1 s (FEV_1_), forced vital capacity (FVC), the FEV_1_/FVC ratio, total lung capacity (TLC), residual volume (RV), alveolar volume (*V*_A_), D_LCOc_, D_LCO_ and the CO transfer coefficient corrected for haemoglobin (*K*_COc_), both as absolute values and as a percentage of predicted (FEV_1_%pred, FVC%pred, FEV_1_/FVC%pred, TLC%pred, RV%pred, D_LCOc_%pred, D_LCO_%pred and K_COc_%pred, respectively) according to height, age and sex (Cotes et al., 1993; Quanjer et al., 1993). Prior to conducting measurements, precise data on the patients' standing height (rounded to the nearest 1 mm), weight (rounded to the nearest 100 g) and haemoglobin (Hb) levels (rounded to the nearest 0.1 mmol/L) were obtained. The measurement of haemoglobin was performed on capillary blood samples using a HemoCue device (Hb 201+; HemoCue, Ängelholm, Sweden).

#### 2.4.2 SPECT

V/Q SPECT scans were performed as part of the initial assessment and performed on a Philips Precedence SPECT CT hybrid scanner (Philips Healthcare, Best Netherlands). Approximately 150 (range, 75−175) MBq ^99m^Tc macro‐aggregated albumin was injected intravenously. After a 140 kVp 5 mm slices 20 mAs low‐dose CT, with a 512 × 512 matrix and a 1.17 mm isotropic pixel size was obtained, the SPECT was acquired (64 × 64 matrix, 64 angles, 12 s per angle in step and shoot mode, 9.3 mm isotropic voxels, energy windows at 190.5 and 140 keV with 20% width), whilst the patient concurrently inhaled from a 600 MBq ^81m^Kr gas generator. The interpretation criteria suggested by the European Association of Nuclear Medicine (EANM) were employed in the analysis and assessment of the obtained results (Bajc et al., 2019). V/Q SPECT images were evaluated to determine whether there were matched ventilation–perfusion defects (= regionally reduced ventilation and perfusion), inversely mismatched ventilation defects (= regionally reduced ventilation with maintained perfusion), mismatched perfusion defects (= regionally reduced perfusion with maintained ventilation) or no defects of any kind. Of note, matched ventilation–perfusion defects and inversely mismatched ventilation defects indicate a ventilatory disturbance, whilst mismatched perfusion defects indicate a vascular disturbance. Furthermore, in those that exhibited mismatched perfusion defects, the severity was assessed by calculating the volume ratio between ventilated and perfused lung tissue on the whole‐lung level through a voxel based analysis of counts. In this analysis, a higher volume ratio thus indicates a greater extent of unperfused (albeit ventilated) lung tissue. All imaging was conducted using IntelliSpace Portal (version 9, Philips Healthcare, Amsterdam, the Netherlands).

#### 2.4.3 Primary endpoint

All‐cause mortality was from first lung function to death or to end of follow‐up. The survival time was censored on the 20 April 2022 (the end of the study period).

### 2.5 Data sources

Lung function data were extracted from a local database (DatGen, version 0.6b) at the Department of Clinical Physiology and Nuclear Medicine at Rigshospitalet in Copenhagen connected to the Department's lung function system (Jaeger MasterScreen PFT pro, CareFusion, Höchberg, Germany). Results of V/Q SPECT were extracted from the Department's radiology information and picture archiving and communication system (RIS/PACS) (Agfa, Mortsel, Belgium). Patient demographic and clinical data (age, sex, PH‐classification, time of death) were extracted from electronic health records (Epic, Verona, WI, USA).

### 2.6 Potential sources of bias

Risk of selection bias ought to be low, as all patients referred to the tertiary PH centre in the study period were included. The PH centre at the Department of Cardiology, Rigshospitalet, handles patients referred from Eastern Denmark, Greenland and the Faroe Islands. The treatment of PH has changed and advanced over the study period and risk may not be independent of time of inclusion. Risk of misclassification of PH in the included patients is considered low, as they undergo extensive diagnostic testing, apart from lung function testing, and V/Q SPECT includes investigations such as right‐heart catheterization, electrocardiography, various blood tests, chest radiography and CT as well transthoracic echocardiography.

### 2.7 Sample size

No formal a priori statistical power calculation was conducted before the study, and sample size was based on available data during the defined study period.

### 2.8 Statistical methods

All statistical analyses were conducted using R statistical software version 4.1.1 (R Foundation for Statistical Computing, Vienna, Austria) within RStudio (version 1.4.1717). A two‐tailed *P *< 0.05 was considered statistically significant. Normally distributed variables are reported as mean (standard deviation (SD)) and mean difference (95% confidence interval (95% CI): lower limit (LL), upper limit (UL)); otherwise, non‐normally distributed data are reported as median and interquartile range (IQR). By examining normality plots and skewness statistics, numerical data were evaluated. To test whether the data met the assumption of a normal distribution, the Shapiro–Wilk test was performed. Bartlett's test was used for testing if data met the assumption of homogeneity of variance. A one‐way ANOVA was performed for D_LCOc_%pred and D_LCO_%pred in different V/Q SPECT defect categories and was followed by Tukey's honestly significant difference *post hoc* test. Spearman's rank correlation coefficient was computed to assess the relationship between diffusing capacity and the SPECT‐based volume ratio. A multivariable Cox regression analyses was performed including a priori selected confounders that were considered important for D_LCOc_%pred, D_LCO_%pred and death: age, sex, height, FEV_1_%pred and haemoglobin. For model comparison, Harrel's concordance index (C‐index) was computed for both D_LCOc_%pred and D_LCO_%pred at 5.7 years of follow‐up. The linear assumption for Cox regression was tested by a cumulative Martingale's residuals test and proportionality assumption by a visual inspection of the cumulative hazards on log‐scale. Goodness of fit (GOF) was used for visualization, and likelihood ratio test statistics with degrees of freedom (df) are reported.

Results are presented as hazard ratio (HR) estimates with 95% confidence intervals. Kaplan–Meier survival curves and log‐rank tests were used to compare univariable survival differences, including patients with a D_LCO_%pred or D_LCOc_%pred ≥45% in accordance with the established PAH subphenotype (Hoeper et al., 2022). In all survival analyses, the time at risk began the day they met at the hospital for the first V/Q SPECT scan.

## 3 RESULTS

### 3.1 Baseline characteristics

**FIGURE 1:**
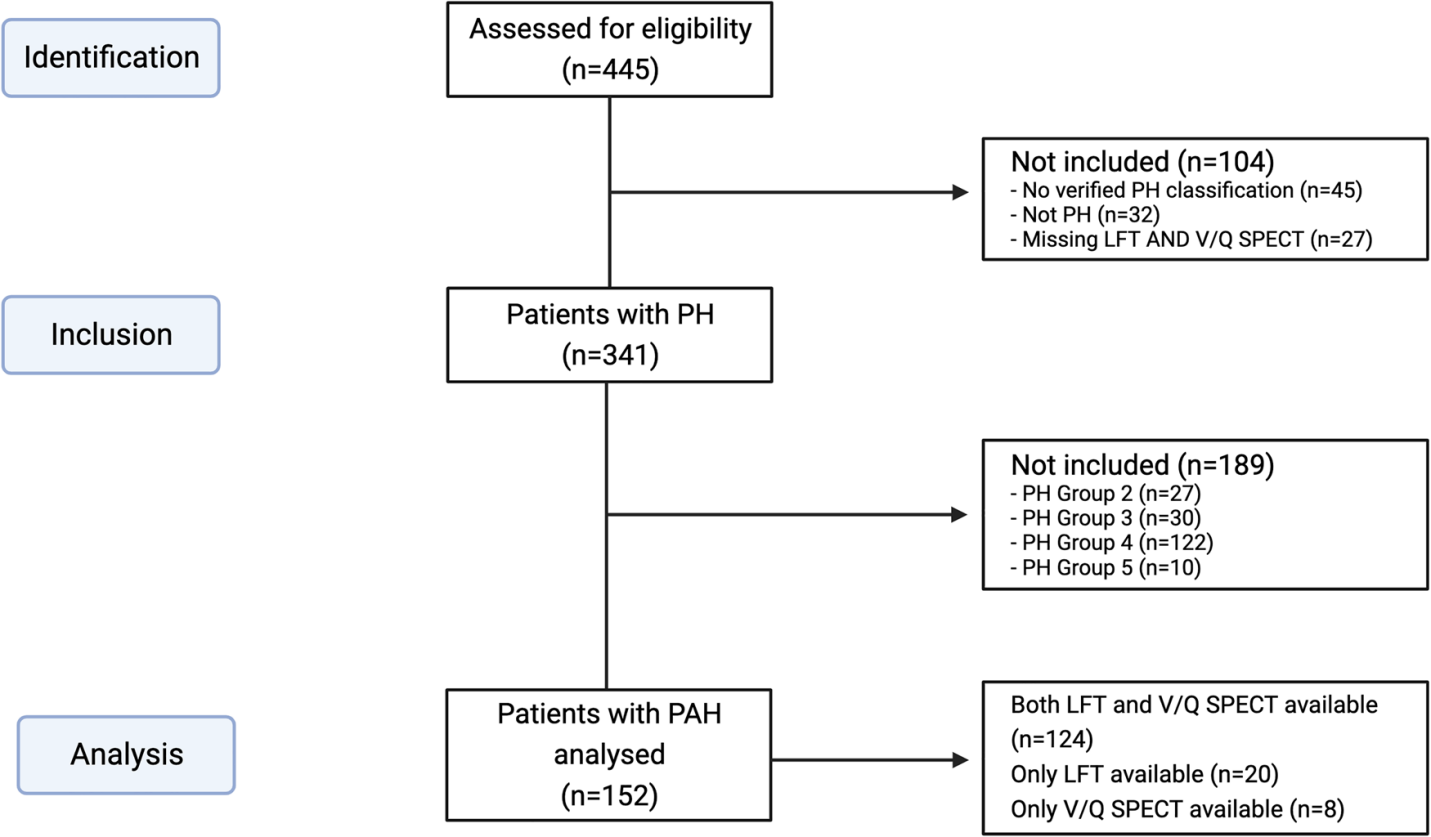
STROBE flow diagram of eligible patients for the current study. Abbreviations: LFT, lung function test; *n*, number of patients; PAH, pulmonary arterial hypertension; PH, pulmonary hypertension; V/Q SPECT, ventilation–perfusion single‐photon emission computed tomography. Created with BioRender.com.

From 445 patients assessed for eligibility, a total of 152 patients with PAH were included in the current study (Figure 1). Baseline characteristics including measured lung function metrics and V/Q SPECT findings are presented in Table 1. Median age (range) at diagnosis was 63 (20–97) and most patients (69.7%) were females.

**TABLE 1:** Baseline data with results from patient characteristics, lung function test and ventilation–perfusion single‐photon emission computed tomography (V/Q SPECT).

Parameter	Value (*n* = 152)
**Patient characteristics**	
Age (years)*	63 (20–97)
Sex (*n* (%))	
Male Female	46 (30%) 106 (70%)
Height (cm) NA	167.32 (8.97) 26
Weight (kg) NA	77.36 (21.60) 26
BMI (kg/m^2^) NA	27.47 (6.59) 26
Hb (mmol/L) NA	8.43 (1.45) 27
**Lung function**	
FEV_1_ (L) NA	2.30 (0.86) 8
FEV_1_%pred NA	84.18 (20.21) 8
FVC (L) NA	3.06 (1.05) 8
FVC%pred NA	92.78 (19.74) 8
FEV_1_/FVC‐ratio NA	0.75 (0.10) 8
FEV_1_/FVC ratio %pred NA	97.82 (12.69) 8
TLC (L) NA	5.12 (1.42) 16
TLC %pred NA	92.26 (29.92) 26
RV (L) NA	1.99 (0.69) 16
RV %pred NA	107.11 (35.91) 26
D_LCOc_ (mmol/min/kPa) NA	4.39 (2.00) 10
D_LCOc_%pred NA	50.51 (18.87) 26
D_LCO_ (mmol/min/kPa) NA	4.34 (2.03) 9
D_LCO_%pred NA	49.56 (19.12) 25
* V*_A_ (litres) NA	4.31 (1.20) 9
K_COc_ (mmol/min/kPa/L) NA	1.03 (0.37) 10
K_COc_%pred NA	66.29 (23.06) 26
**V/Q SPECT**	
Mismatch	48 (32%)
Matched	62 (41%)
Inverse mismatch	24 (16%)
No defect	31 (20%)
NA	20 (13%)

*Note*: Values are presented as means (SD) unless otherwise specified. *Median (range). Matched: regionally reduced ventilation and perfusion; mismatch: regionally reduced perfusion with maintained ventilation; inverse mismatch: regionally reduced ventilation with maintained perfusion; no defect: no significant defect detected on V/Q SPECT.

Abbreviations: BMI, body mass index; D_LCO_, pulmonary diffusing capacity for carbon monoxide; D_LCOc_, pulmonary diffusing capacity for carbon monoxide corrected for haemoglobin; FEV_1_, forced expiratory volume in 1 s; FVC, forced vital capacity; Hb, haemoglobin; *K*_COc_, transfer coefficient of the lung for carbon monoxide corrected for haemoglobin; *n*, number of patients; NA, not available; RV, residual volume; TLC, total lung capacity; V/Q SPECT, ventilation–perfusion single‐photon emission computed tomography; *V*_A_, alveolar volume.

### 3.2 Diffusing capacity and mortality

**FIGURE 2:**
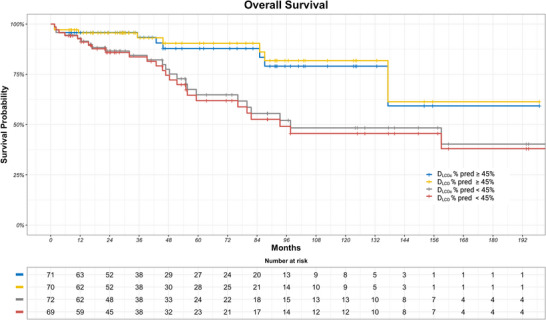
Kaplan–Meier survival curve according to diffusing capacity of the lung for carbon monoxide corrected for haemoglobin as percentage of predicted (D_LCOc_%pred, blue line) and diffusing capacity of the lung for carbon monoxide as percentage of predicted (D_LCO_%pred, yellow line) ≥45% of predicted compared to D_LCOc_%pred (grey line) and D_LCO_%pred (red line) < 45% of predicted. Log rank test, *P* = 0.02 for D_LCOc_%pred ≥ 45% compared to D_LCOc_%pred < 45%. Log rank test, *P* = 0.003 for D_LCO_%pred ≥ 45% compared to D_LCO_%pred < 45%. Abbreviations: D_LCO_, diffusing capacity for carbon monoxide; D_LCOc_, diffusing capacity for carbon monoxide corrected for haemoglobin.

In total, 35 patients (23%) died during a mean follow‐up of 5.7 years. In Kaplan–Meier analyses dichotomising D_LCOc_%pred based on the mean value, there was difference in survival between D_LCOc_%pred ≥ 45% and D_LCOc_%pred < 45% (log rank, *P* = 0.02) (Figure 2). The same was observed for D_LCO_%pred ≥ 45% and D_LCO_%pred < 45% (log rank, *P* = 0.003) (Figure 2). No difference was seen in respect to survival between D_LCOc_%pred ≥ 45% and D_LCO_%pred ≥ 45%.

In unadjusted Cox analysis, D_LCOc_%pred (HR per 1 SD = 0.427; 95% CI: 0.255–0.713; *P* = 0.0012) and D_LCO_%pred (HR per 1 SD = 0.494; 95% CI: 0.318–0.768; *P* = 0.0017) were similarly associated with all‐cause mortality. The similar HRs remained after adjustment for age, sex, height, FEV_1_%pred and haemoglobin; for D_LCOc_%pred each percentage increase in D_LCOc_%pred was associated with a 3.3% decrease in hazard of death (HR = 0.967; 95% CI: 0.938–0.997; *P* = 0.0313) with a 47.1% decrease (HR = 0.529; 95% CI: 0.296–0.944) per 1 SD increase, and for D_LCO_%pred each percentage increase was associated with a 2.9% decrease in hazard of death (HR = 0.971; 95% CI: 0.943–0.999; *P* = 0.0488) with a 43.3% decrease (HR = 0.567; 95% CI: 0.327–0.997) per 1 SD increase. The C‐index of the multivariate Cox regression analysis with D_LCOc_%pred and D_LCO_%pred was similar at 0.71 and 0.72, respectively (*P* = 0.85). Visualisation for GOF showed similarity between models with D_LCOc_%pred and D_LCO_%pred. Likelihood ratio test statistics for D_LCOc_%pred (20.1 on 6 df, *P* = 0.003) and D_LCO_%pred (18.5 on 6 df, *P* = 0.005) were also nearly similar, although slightly favouring D_LCOc_%pred.

### 3.3 V/Q SPECT and mortality

**FIGURE 3:**
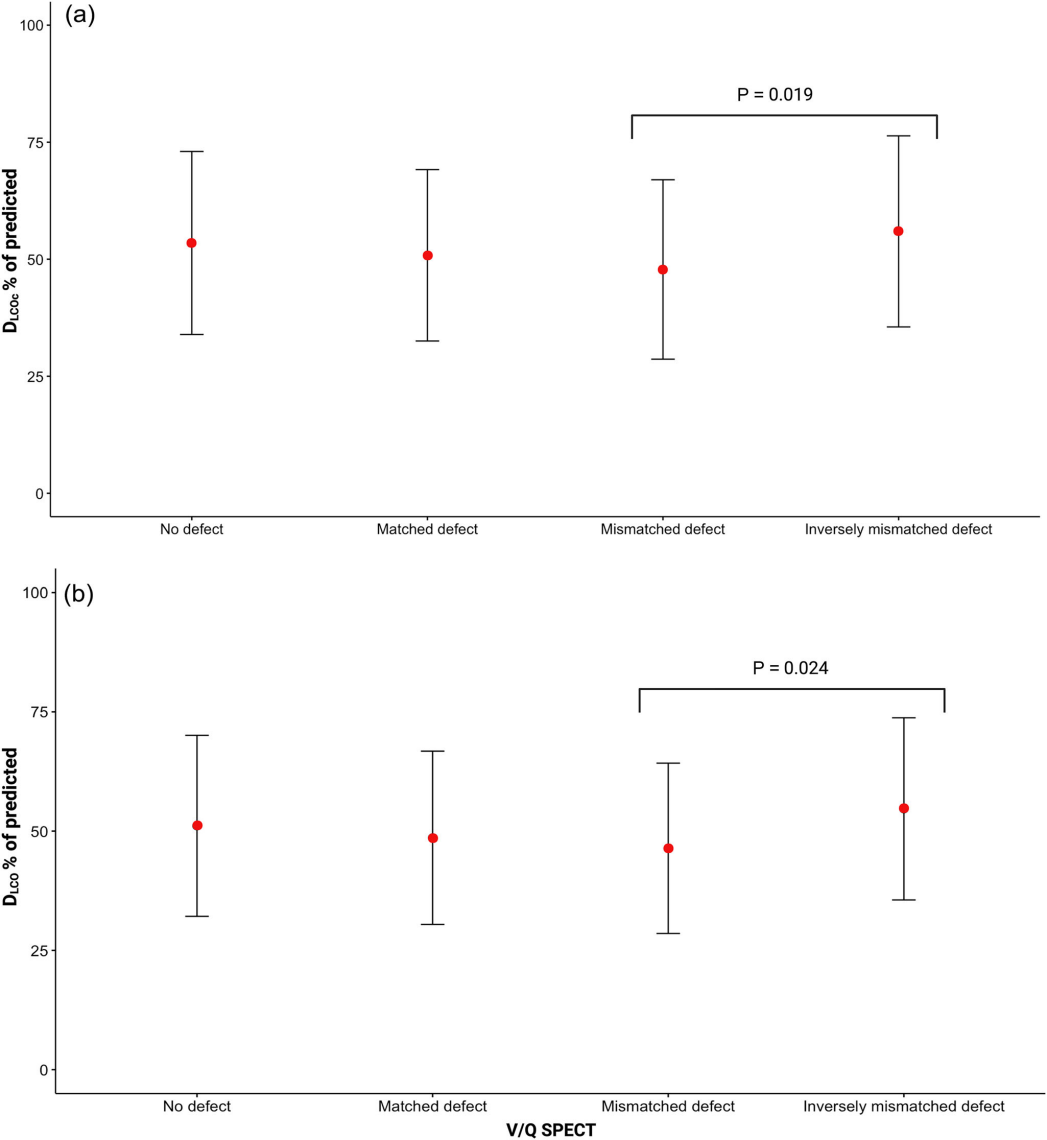
Visual presentation of the relationship between D_LCOc_% of predicted (a) and D_LCO_% of predicted (b) and different defects on V/Q SPECT. Mean values presented as a red circle with SD error bars. A one‐way ANOVA was performed for D_LCOc_% and D_LCO_% in different V/Q SPECT defect categories and was followed by a Tukey's honestly significant difference *post hoc* test. A two‐tailed *P *< 0.05 was considered statistically significant. For both D_LCOc_% and D_LCO_%, there were only significant difference between ‘Inversely mismatched defect’ and ‘Mismatched defect’ (*P* = 0.019 and *P* = 0.024, respectively). Abbreviations: D_LCO_, diffusing capacity for carbon monoxide; V/Q SPECT, ventilation–perfusion emission computed tomography.

Patients with different types of V/Q SPECT defects exhibited differences in their D_LCOc_%pred and D_LCO_%pred values (*P* = 0.020 and 0.018). More specifically, patients with inversely mismatched ventilation defects had the highest D_LCOc_%pred at 62.67 (20.52)% and D_LCO_%pred 62.21 (20.05)%, whilst those with mismatched perfusion defects had the lowest mean values of 40.21 (15.85)% and 40.01 (15.99)%, respectively. Patients with matched ventilation–perfusion defects had a D_LCOc_%pred of 48.39 (17.27)% and D_LCO_%pred of 46.26 (18.27)%, whereas patients with both matched and mismatched defects had a D_LCOc_%pred and D_LCO_%pred of 54.7 (19.63)% and 54.91 (20.61)%, respectively. Patients with no defects on V/Q SPECT had a D_LCOc_%pred of 52.98 (19.74)% and D_LCO_%pred of 51.10 (18.97)%. Of note, D_LCOc_%pred and D_LCO_%pred values were higher in patients with inversely mismatched ventilation defects when compared with mismatched perfusion defects (*P* = 0.019 and 0.024, respectively; see Figure 3). In those with mismatched perfusion defects, the correlational analyses between D_LCOc_%pred and SPECT‐based volume ratio showed *r* = −0.32 (*P* = 0.03), with *r* = −0.29 (*P* = 0.06) for D_LCO_%pred versus SPECT‐based volume ratio. None of the V/Q SPECT findings had a detectable impact on univariable HRs for all‐cause mortality (Table 2).

**TABLE 2:** Results from univariable Cox regression for all‐cause mortality with different kinds of V/Q SPECT pattern.

V/Q SPECT	HR	95% CI	*P*
Matched defect	0.77	0.38–1.50	0.45
Inversely mismatched defect	0.70	0.20–2.30	0.57
Mismatched defect	1.1	0.57–2.30	0.70
Any defect	0.66	0.32–1.40	0.29
No defect at all	1.5	0.72–3.20	0.27

Any defect: any kind of different defects on V/Q SPECT; inversely mismatched defect: regionally reduced ventilation with maintained perfusion; matched defect: regionally reduced ventilation and perfusion; mismatched defect: regionally reduced perfusion with maintained ventilation; no defect: no significant defect detected on V/Q SPECT. Abbreviations: V/Q SPECT, ventilation–perfusion single‐photon emission computed tomography.

## 4 DISCUSSION

The main findings of the present study are that low D_LCOc_%pred and D_LCO_%pred are similarly associated with poor survival rate, independently of other variables from the lung function test measured at baseline in patients with verified PAH. Hence, the hazard for all‐cause mortality decreased by 3.3% and 2.9% for each percentage point increase in D_LCOc_%pred and D_LCO_%pred, respectively. Ancillary findings on V/Q SPECT scans were differently associated with D_LCOc_%pred and D_LCO_%pred but did not seem to inform prognosis further.

The comparative prognostic impact of D_LCOc_%pred versus D_LCO_%pred has to our knowledge not previously been investigated, but our results do largely align with those from a recent study in which the hazard of mortality was found to decrease by 6% for each percentage increase in D_LCOc_%pred in patients with PAH (Diamanti et al., 2022). Likewise, D_LCO_ has also been reported to predict poorer event‐free survival regardless of the presence of concurrent lung disease (Chandra et al., 2010; Kang et al., 2017; Lewis et al., 2020; Szturmowicz et al., 2016; Trip et al., 2013), and was highlighted as the greatest predictor of mortality in a recent prospective cohort study of PAH patients, with HR decrease similar to that reported in the present study of a 2.6% for each percentage increase in D_LCO_ (Stadler et al., 2019). Similar findings have been reported on the negative prognostic value of low D_LCO_ in PAH patients (Benza et al., 2010; Lee et al., 2012; Szturmowicz et al., 2016). Overall, this study, in alignment with previous studies, has highlighted the significance of baseline D_LCO_ and D_LCOc_ in PAH (Diamanti et al., 2022). Collectively, ours and these previous findings supports the existence of a clinically relevant PAH subphenotype with low D_LCO_ or D_LCOc_ (Hoeper et al., 2022), as well as the use of D_LCO_ and D_LCOc_ in PAH risk stratification scores as currently recommended in the so‐called REVEAL 2.0 risk assessment tool (Benza et al., 2019).

D_LCOc_ quantifies the lungs' efficiency in transferring gas from the alveolar region to blood, independently of haemoglobin levels. Given that it is affected by a wide variety of factors, its robust prognostic capability may be attributed to its sensitivity to a range of factors such as smoking with resultant emphysema, causing reduced D_LCOc_ due to alveolar and capillary destruction. This reduces both the alveolar‐capillary surface area available for diffusion and the pulmonary capillary blood volume. Also, smoking itself could potentially contribute to damage in the pulmonary vasculature that is known to precede manifest emphysema (Alford et al., 2010; Peinado et al., 2008; Seimetz et al., 2011). In addition, conditions such as low cardiac output and right heart dysfunction indirectly affect D_LCOc_ by influencing pulmonary haemodynamics and, subsequently, gas exchange efficiency. Notably, alterations in the diffusing capacity can be attributed to several factors including modifications in the area available for gas exchange, alterations in the integrity of the alveolar–capillary membrane, ventilation/perfusion mismatch and involvement of pulmonary capillaries (Fritz & Smith, 2016; Trip et al., 2013). PAH itself may cause remodelling of the alveolar–capillary membrane and reduce capillary blood volume that could account for the effects on gas transfer (Guazzi, 2008), reflecting that progressive pulmonary vascular disease affect D_LCOc_ negatively. Hence, the observed association between mortality and low levels of D_LCOc_ in PAH likely reflects fundamental pathophysiological processes within the pulmonary vasculature with a concomitant reduction in pulmonary capillaries available for perfusion, thus causing a reduction in diffusing capacity (Suda et al., 2017).

Our findings indicate a substantial disparity in the levels of both D_LCOc_%pred and D_LCO_%pred when comparing PAH patients exhibiting inversely mismatched defects as opposed to those with mismatched perfusion defects. PAH patients with mismatched perfusion defects had the lowest values of D_LCOc_%pred and D_LCO_%pred, likely reflecting the underlying vascular remodelling. Furthermore, the similarly weak negative correlations between the diffusing capacity measures and the SPECT‐based volume ratio in those with mismatched perfusion defects, which nevertheless supports the contention that these perfusion disturbances affect pulmonary gas exchange. On the contrary, patients with inverse mismatch exhibited the highest values of D_LCOc_%pred and D_LCO_%pred, even though the V/Q SPECT finding indicates the presence of a ventilatory disturbance with attenuated hypoxic pulmonary vasoconstriction, which should cause a further impairment of pulmonary gas exchange. The underlying mechanism of this nevertheless remains elusive.

When investigating the clinical significance of ancillary findings on V/Q SPECT performed to exclude CTEPH, our findings suggest that no specific type of V/Q SPECT defects in PAH patients were associated with higher hazard of mortality. However, we found that mismatched defects were significantly associated with a low D_LCOc_%pred and D_LCO_%pred. Of note, in accordance with the routine diagnostic workup, the presence of pulmonary thromboembolic disease had been ruled out by CT angiography in these cases. Thus, the observed perfusion defects were typically part of a characteristic ‘patchy’ perfusion pattern characteristic of PAH (Chan et al., 2018; Wang et al., 2019), most probably reflecting disease‐specific loss and obliteration of the microvasculature (Humbert et al., 2019), which also provides a putative mechanistic link to the associated low D_LCOc_%pred and D_LCO_% pred. The finding that these mismatched perfusion defects are nor associated with mortality differ from those in a previous study on 75 PAH patients, in which the presence of mismatched perfusion defects was associated with a higher all‐cause mortality rate with a HR of 5.63 (Chan et al., 2018) compared to those with no defects. These results suggest that V/Q SPECT may offer utility beyond its conventional role in excluding CTEPH, but these findings could not be directly replicated in our study. Overall, the contrasting findings warrant further exploration to ascertain the precise mechanisms and underlying factors contributing to the observed association between global perfusion defects and adverse clinical outcomes in PAH patients.

There are several limitations to this study. The study was conducted in a single centre with a retrospective cohort design and data were missing in some patients. Various clinical data from the DAN‐PH database were not included in this study, and our study is thus not suitable for detecting additional potentially significant prognostic factors associated with survival. However, all lung function tests and V/Q SPECT were performed at our department, which contributes to decreased variability. Overall, our analytical methods did not afford us the opportunity to investigate the underlying pathophysiological mechanisms responsible for the observed reduced diffusing capacity. However, we were able to assess the severity of perfusion defects and investigate the relationship with diffusing capacity and additional risk stratification.

All‐cause mortality was our singular outcome, which is accompanied by a lack of specificity. All‐cause mortality constitutes a measurement of the comprehensive risk associated with mortality from any aetiology, yet it fails to offer discernment into the precise causes of death. This inherent absence of specificity imposes limitations upon the comprehension of disease patterns and the formulation of interventions that could be targeted towards specific aetiologies. However, all‐cause mortality remains a clinically important end point with no competing risk influencing this in our study. We included PAH patients diagnosed between 2011 to 2020. This is a broad inclusion period and several changes in PAH patients’ characteristics but also medication treatment have occurred during the study timeline. Even though adjusting for time of inclusion did not change our conclusion, we could not rule out that the PAH patients do not completely represent the incident cases of PAH seen today. Moreover, we did not discriminate between the specific subgroups of PAH in our analysis. As patients underwent extensive diagnostic testing to classify PH, subjects in our cohort did not have any cardio‐pulmonary co‐comorbidities that would interfere with other PH classifications, but more specific co‐morbidities were inaccessible.

Lastly, we could not elaborate further on the directionality of the association between V/Q defects and values for diffusing capacity. Notably, PAH often involves altered right ventricle function, significantly impacting both pulmonary and cardiac dynamics. As a complex interplay between pulmonary mechanics, gas exchange and cardiac function is found in PAH, we could thus speculate that low diffusing capacity in those with mismatched perfusion defect could be attributed to low cardiac output, but this information was not available due to the retrospective design of our study.

## 5 CONCLUSION

In patients with PAH, both a low D_LCOc_%pred and a low D_LCO_%pred obtained at the initial diagnostic workup are associated with an increased hazard of all‐cause mortality. Whilst these two metrics may differ between PAH patients with different abnormalities on V/Q‐SPECT, the haemoglobin correction does not in itself appear to offer any additional benefit in terms of prognostic stratification. Thus, in the clinical setting, both D_LCOc_ and D_LCO_ should be considered equal for PAH risk assessment.

## AUTHOR CONTRIBUTIONS

Milan Mohammad: Data collection, data analysis, data interpretation, figures, first draft, revisions. Jacob P. Hartmann: Data analysis, data interpretation, figures, first draft, revisions. Jørn Carlsen: Conception, data interpretation, revisions. Anders M. Greve: Data analysis, data interpretation, revisions. Ronan M. G. Berg: Conception, design, data collection, data interpretation, revisions, supervision. Jann Mortensen: Conception, design, data interpretation, revisions, supervision. All persons designated as authors qualify for authorship, and all those who qualify for authorship are listed. All authors approved the final version of the manuscript and agree to be accountable for all aspects of the work in ensuring that questions related to the accuracy or integrity of any part of the work are appropriately investigated and resolved. Ronan M. G. Berg is guarantor of this work and accepts full responsibility for the work and the conduct of the study, had access to the data and controlled the decision to publish.

## CONFLICT OF INTEREST

None of the authors have conflict of interest to declare.

## Data Availability

The data underlying our findings can be shared upon reasonable request directed to the corresponding author.
